# Effect of Different Primers on Shear Bond Strength of Base Metal Alloys and Zirconia Frameworks

**DOI:** 10.3390/polym16050572

**Published:** 2024-02-20

**Authors:** Marco Dederichs, Zaid Badr, Stephanie Viebranz, Steffen Schroeter, Christoph-Ludwig Hennig, Anne-Sophie Schmelzer, Arndt Guentsch

**Affiliations:** 1 Policlinic of Prosthetic Dentistry and Material Science, Center for Dental Medicine, Jena University Hospital, D-07743 Jena, Germany; 2 Technological Innovation Center, Department of General Dental Sciences, Marquette University School of Dentistry, Milwaukee, WI 53201-1881, USA; 3 Department of Orthodontics, Center for Dental Medicine, Jena University Hospital, D-07743 Jena, Germany; 4 Department of Surgical and Diagnostic Sciences, Marquette University School of Dentistry, Milwaukee, WI 53201-1881, USA

**Keywords:** base metal alloy, zirconia, adhesion, primer, 10-MDP, composite

## Abstract

Ensuring a secure bond between a framework structure and layering composite resin veneer is essential for a long-lasting dental restoration. A variety of primer systems are available to facilitate the adhesive bonding. Nevertheless, the growing preference for efficiency and simplicity in dentistry has made the one-bottle universal primers a desirable option. This study aims to compare the effectiveness of universal primers on the shear bond strength (SBS) of base metal alloy (BMA) and zirconia to layering composite resin. Each 160 BMA and zirconia 20 × 10 × 5 mm test specimen was fabricated. Eight different primers (SunCera Metal Primer, Metal Primer Z, Reliance Metal Primer, Alloy Primer, MKZ Primer, Monobond Plus, ArtPrime Plus, and Clearfil Ceramic Primer Plus) were applied to 20 specimens in each group. Subsequently, a 5 × 2 mm composite resin build-up was applied. SBS tests were performed after 24 h of water storage and after thermocycling (25,000 cycles, 5–55 °C). On BMA, after water storage for 24 h, the bond strength values ranged from 26.53 ± 3.28 MPa (Metal Primer Z) to 29.72 ± 2.00 MPa (MKZ Primer), while after thermocycling, bond strength values ranged from 25.19 ± 1.73 MPa (MKZ Primer) to 27.69 ± 2.37 MPa (Clearfil Ceramic Primer Plus). On a zirconia base, after 24 h, the bond strengths values ranged from 22.63 ± 2.28 MPa (Reliance Primer) to 29.96 ± 2.37 MPa (MKZ Primer) and from 23.77 ± 3.86 MPa (Metal Primer Z) to 28.88 ± 3.09 MPa (Monobond Plus) after thermocycling. While no significant difference in bond strength was found between the primers on the BMA base, five primer combinations differed significantly from each other on zirconia (*p* = 0.002–0.043). All primers achieved a bond strength greater than 23 MPa on both framework materials after thermocycling. Thus, all primers tested can be applied to both framework materials with comparable results.

## 1. Introduction

For decades, cobalt-chrome-based alloys, also known as base metal alloys (BMAs), have been regarded in dental technology and dentistry as suitable framework bases for a wide range of dental restorations [1]. As one of the most robust framework materials, BMA alloys can be used almost universally. With a Young’s modulus of about 200–220 GPa and a tensile strength of up to 1000 MPa, this class of materials can be used to manufacture both removable partial dentures and wide-span fixed dental prosthesis [2]. Due to their wide range of applications BMAs continue to be a fundamental basis for the manufacture of dental restorations [1]. From the patient’s point of view, however, the attitude towards metal often has a great influence on the acceptance of this material group. There is an increasing desire for completely metal-free restorations, even for extended dental restorations [1,3]. Zirconia has not been in use for quite as long as BMAs, but since the 1990s, 3 mol% yttria-partially stabilized zirconia (3Y-PSZ) has gained increasing popularity in dentistry as a framework material [4]. Zirconia has become a well-established and indispensable material for the production of fixed dental prostheses. With a Young’s modulus of up to 210 GPa and a flexural strength of up to 1600 MPa, zirconia and BMAs cover a wide range of indications, especially in the planning and manufacture of fixed dental prostheses [5]. Especially in the field of implant-supported superstructures, the desire for metal-free restorations is increasing [3].

While a tooth-colored veneering on BMA frameworks is mandatory in the esthetically demanding zone, modern zirconia can achieve acceptable results even in the esthetic zone through increased translucency or internal layering [6,7,8]. In cases where high esthetic demands are desired, a ceramic veneer is usually applied on both material bases [4,6]. However, esthetic ceramic veneers are not always the veneering material of choice. For example, in telescopic prosthetics, composite resin veneers are the material of choice due to the generated internal stresses during insertion and removal of the prosthesis. These internal stresses could pose an increased risk of cracks or fractures of the veneering ceramic. Therefore, in cases where stress tolerance is crucial, composite resin veneers are often preferred due to their superior ability to withstand such stresses. This characteristic makes them a preferred material for use in telescopic prosthetics [9,10,11,12]. Composite resin veneers can also be used with zirconia frameworks [13,14]. For example, composite resin veneering of implant-supported zirconia frameworks is a promising combination that can meet the desired esthetic demands while reducing the weight and cost of the prosthesis [13,15]. In addition, composite resin veneers offer a significant advantage over ceramic veneers in cases of chipping. In such events, composite resin veneers can be predictably repaired intraorally, which could prevent or delay the complete renewal of the prosthesis [16,17,18].

To achieve a secure and stable bond between the framework and veneering composite resin, the use of chemical coupling agents, such as those found in primers, is essential [16,19,20,21]. While ceramic fusing materials form stable ionic bonds with the framework base, bonding to the oxidized layer [22], the adhesion mechanism between composite resins and the framework base is different. The composition of these primers varies based on the framework material and specific application. These primers typically contain either bi-functional or multi-functional monomers. The functional end of these monomers facing the metal or ceramic surface may contain sulfur-containing or dithiooctanoate-containing functional groups to bind to noble metals [16,20,23]. On the other hand, phosphate functional groups are essential when bonding to BMAs, ceramics or even to tooth enamel and dentin [16,20,23]. The other functional end of these primer-monomers has a carboxylated end, which enables chemical bonding to methacrylate-containing adhesives or composite resins. Both functional groups are linked to each other by connecting molecules that usually contain carboxylates [23,24].

The one-bottle primers quickly became very popular due to their ease of handling when compared with the classically multi-step primers. This led to the emergence of a number of primers in the market in recent decades [23]. While the major challenge of the early generation primers was to establish a reliable bond between metals and composites due to the different affinity to adhesive monomers [20,25], the introduction of zirconia in dentistry presented even more new challenges to the existing primers [16]. Following the demand of dental technology, new primers were developed to be used universally on both metals and zirconia. The demand for new primers extends beyond the dental laboratory to clinical settings, when repairing a chipped ceramic veneer. Accordingly, there is a great desire for universal primers requiring as few steps as possible that can be used in as many applications as possible [23].

The present exploratory study addresses this need and aims to reveal whether the one-fits-all concept can be applied to a selection of popular primers. Therefore, the aim of this study was to apply various known primers to both BMA and zirconia bases and to evaluate and assess the bond between these bases and a veneering composite via shear bond strength test. The null hypotheses were the following: (1) There are no mean differences in bond strength between the base metal alloy and the veneering composite resin among different primers. (2) There are no mean differences in bond strength between the zirconia base and the veneering composite resin among different primers. (3) There are no mean differences in bond strength between the base metal alloy and the zirconia base when different primers are used.

## 2. Materials and Methods

**Figure 1:**
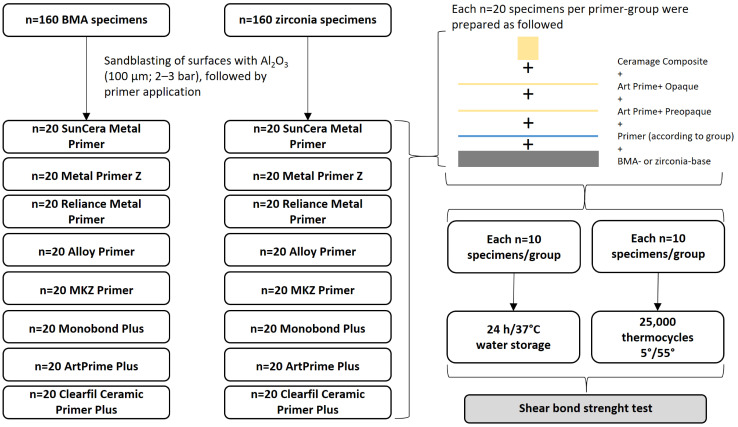
Flow chart of the experimental setup. *N* = 160 specimens of each framework material (BMA or zirconia) were used for the experimental setup. Each *n* = 20 specimens of each base material was primed with one of the primer agents according to manufacturer’s recommendation. Then ArtPreOpaque and ArtOpaque were applicated, followed by a shear-buildup made from Ceramage composite. Half of the specimens (*n* = 10) underwent a shear bond strength test after 24 h of water storage, while the other half (*n* = 10) of specimens went through thermocycling first, before facing shear bond strength tests.

A total of *n* = 160 BMA specimens and *n* = 160 zirconia specimens were prepared for the tests. A schematic overview of the specimen preparation is shown in Figure 1. The materials and primers used for the buildup structure are presented in Table 1 and Table 2. The preparation of the BMA specimens and the zirconia specimens was carried out in the same manner. All BMA (Heraenium CE (Batch No.: 12933, Kulzer, Hanau, Germany)) and 3Y-PSZ zirconia (Batch No.: DTCJU, Katana Zirconia HT, Kuraray-Noritake, Nagoya, Japan) test specimens (specimen base size 20 × 10 × 5 mm) were first airborne-particle abraded with corundum (Al_2_O_3_, 110 µm, under 3 bars for BMA specimens and 2 bars for zirconia specimens), at a blasting angle of 45°. The surfaces were then cleaned with oil-free compressed air for 5 s at 0.2 MPa (2 bars). A total of eight different primers were investigated (SunCera Metal Primer, Merz Dental, Luetjenburg, Germany; Metal Primer Z, GC, Tokyo, Japan; Reliance Metal Primer, Reliance Orthodontic Products Inc., Itasca, IL, USA; Alloy Primer, Kuraray-Noritake, Nagoya, Japan; MKZ Primer, bredent, Senden, Germany; Monobond Plus, IvoclarVivadent, Schaan, Lichtenstein; ArtPrime Plus, Merz Dental, Luetjenburg, Germany; and Clearfil Ceramic Primer Plus, Kuraray-Noritake, Nagoya, Japan). For each primer, *n* = 20 test specimens per framework material were coated with one type of primer. For this purpose, the respective primer was applied to the air-abraded surface and distributed with a microbrush, following the manufacturer’s recommendations (Table 2). After the individual reaction time (see Table 2), the priming opaque ArtPreOpaque Plus (Merz Dental, Luetjenburg, Germany) was applied uniformly to all specimens. This was applied in a thin layer and evenly distributed using a firm short-haired brush and then photopolymerized for 180 s in the bre.LuxPowerUnit 2 polymerization unit (bredent, Senden, Germany). Subsequently, the opaque ArtOpaque Plus (Merz Dental, Luetjenburg, Germany) was applied. The opaque was applied in a thin layer and distributed evenly twice with a firm short-hair brush. Between the two opaque layers and after the second opaque application, light-curing was performed in the bre.LuxPowerUnit 2 polymerization unit (bredent, Senden, Germany) for 180 s each. A brass ring (Ø 5 mm, height 2 mm) was then placed centrally on each of the prepared surfaces and filled with a Ceramage light curing crown and bridge composite (Shofu, Kyoto, Japan) and photopolymerized with the bre.LuxPowerUnit 2 (bredent, Senden, Germany) for 180 s. After curing, the metal ring was gently removed. This was followed by final light curing for 180 s using the bre.LuxPowerUnit 2 (bredent, Senden, Germany).

The prepared test specimens were first stored in water for 24 h at 37 °C. After 24 h, each test series consisting of 20 specimens was divided randomly. *N* = 10 specimens were immediately subjected to the shear bond strength test, while the second half (the remaining *n* = 10 specimens) were subjected to artificial aging simulation by means of thermocycling. This was performed in a thermocycler (SN:A1009, SD Mechatronik GmbH, Feldkirchen-Westerham, Germany) under 25,000 thermocycles in water baths between 5 °C and 55 °C, in accordance with DIN EN ISO 10477 [26]. The shear bond strength testing after 24 h and after thermocycling were performed using the Z005 universal testing machine (ZwickRoell GmbH & Co. KG, Ulm, Germany) at room temperature. The crosshead speed was set to 1 mm/min. Fracture surfaces were evaluated with a Zeiss Axiotech microscope under up to 50× magnification (Zeiss, Jena, Germany). The tested specimens were classified according to their fracture pattern. Therefore, a measuring eyepiece with crosshair projection and micrometer scaling was used for the quantitative classification of the fracture patterns. Three possible fracture levels were defined: at the level of the substrate base, at the level of the opaque and at the level of the composite structure. In addition, two types of mixed fractures were defined: a mixed fracture at substrate/opaque level or at opaque/composite level. The fracture pattern were declared as ‘adhesive fracture’ if remnants of the opaque or composite resin build-up, respectively, covered less than 25% of the bonding surface. They were declared as ‘cohesive fracture’ if remnants of the opaque or bonded composite resin build-up, respectively, covered more than 75% of the bonding surface. In cases were remnants of the opaque or bonded composite build-up covered 25–75% of the bonding surface, they were declared as ‘mixed fracture’. In this case, a distinction was also made as to whether the mixed fracture exposed 25–75% of the substrate base (BMA or zirconia) or whether the mixed fracture occurred primarily in the opaque/composite build-up area.

**Table 2:** Technical data of primers used for specimen preparation.

Primer	Functional Components	Scope of Application	Application	Batch No.
SunCera Metal Primer (Merz Dental, Luetjenburg, Germany)	phosphonic acid monomer, thiocticacid monomer, acetone	metal, PEEK	Air-particle abrasion (50–110 µm), clean with oil-free compressed air, apply the primer with a brush, leave for 10 s.	051706
Metal Primer Z (GC, Tokyo, Japan)	10-MDP, MDTP	metal, zirconia	Air-particle abrasion, clean with oil-free compressed air, apply a thin layer on bonding surface, allow to dry.	1810161
Reliance Metal Primer (Reliance Orthodontic Products Inc., Itasca, IL, USA)	4-META [27,28]	metal	Air-particle abrasion of surface, rinse and dry thoroughly, application of 1 coat of primer on surface, leave for 30 s.	182171
Alloy Primer (Kuraray-Noritake, Nagoya, Japan)	10-MDP, VBATDT	metal	Air-particle abrasion, clean, apply a thin coating on the surface, leave for 5 s reaction time.	580093
MKZ Primer (bredent, Senden, Germany)	MPS, 10-MDP	metal, ceramic, zirconia	Air-particle abrasion (metal framework: 110 µm, 3–4 bar/ceramic or zirconia: 110 µm, 2 bars), impurities can be removed with alcohol and a clean brush, no cleaning with steam jet, application of primer and rest for 30 s for evaporation.	471448
Monobond Plus (IvoclarVivadent, Schaan, Lichtenstein)	Alcohol solution of silane methacrylate, 10-MDP [29], sulfide methacrylate	universal primer	Air-particle abrasion, if necessary ultrasonic cleaning of restoration for about 1 min, rinse with water spray and dry with oil-free compressed air, apply a thin coat of primer with a brush, rest for reaction time for 60 s, disperse any remaining excess with a strong stream of air.	W10892
ArtPrime Plus Metal Primer (Merz Dental, Luetjenburg, Germany)	Acetone, phosphonic acid monomer, thioctic acid	universal metal primer	Air-particle abrasion (50–110 µm), clean with water and dry with oil-free compressed air, apply the primer with a brush, leave for 10 s.	011918
Clearfil Ceramic Primer Plus (Kuraray-Noritake, Nagoya, Japan)	MPS,10-MDP, ethanol	Universal primer	Air-particle abrasion (30–50 µm, 1–4 bars according to framework material), ultrasonic cleaning for 2 min, dry with oil-free compressed air, apply the primer with a brush, dry the surface with mild oil-free air flow.	BA0031

MDTP = 10-methacryloyloxydecyl-dihydrogen-thiophosphate; 4-META = 4-methacryloxyethyl trimellitateanhydrideVBATDT = 6-(4-vinylbenzyl-n-propyl)amino-1,3,5-triazine2,4-dithione; 10-MDP = 10-Methacryloyloxydecyl-dihydrogen-phoshate; MPS = 3-Methacryloxypropyl-trimethoxysilane.

Bond strength values from shear bond strength testing were collected in an Excel sheet (Microsoft, Redmond, WA, USA) and statistically evaluated using SPSS Statistics 26.0 (SPSS Inc., Chicago, IL, USA). To analyze the differences between shear bond strength values after 24 h of storage compared to those after thermocycling, as well as to evaluate differences in shear bond strength values between BMA versus zirconia bases, the Mann–Whitney U test was used. To reveal potential significant differences between the different primers, the Kruskal–Wallis test was used. The level of significance was set at α = 0.05. A power analysis was performed to calculate the power based on the shear strength values for one of the primers, given a sample size of *n* = 10, shear bond strength values (MPa) between the zircon base and the veneering composite of 28.12 ± 2.03 MPa after 24h water storage and 23.77 ± 3.86 MPa after thermocycling, and a significance level of 0.05 (free statistical software G*Power 3.1.9.7). The calculated power of this study was 90.52%.

## 3. Results

### 3.1. Adhesive Bond on BMA Base

**Figure 2:**
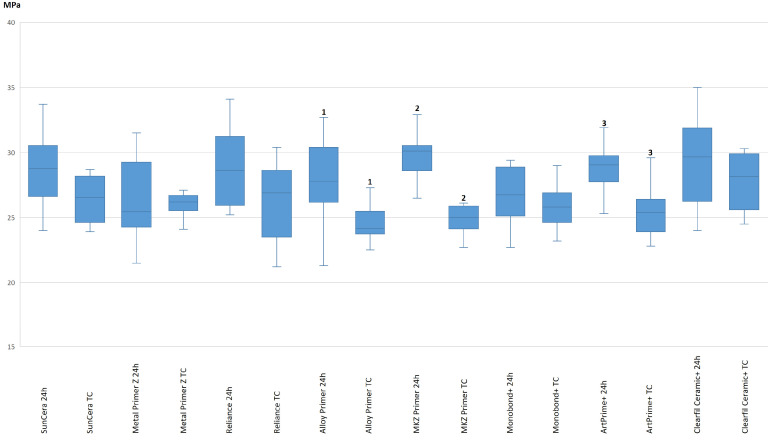
Comparison of the adhesive bond strength on a BMA base (24 h versus thermocycling (TC)). Significant differences within a primer (24 h vs. TC) are indicated by Arabic numerals. Equal numbers indicate significant differences.

Before thermocycling (24 h values), the shear bond strength values were found to be in the range of 26.53 ± 3.28 MPa (Metal Primer Z) to 29.72 ± 2.00 MPa (MKZ Primer). After 25,000 thermocyclings, the bond strength values were in a similarly close range, from 25.19 ± 1.73 MPa (MKZ Primer) to 27.69 ± 2.37 MPa (Clearfil Ceramic Primer Plus). No significant differences were found between the different primers (Kruskal–Wallis test *p* = 0.144). However, within the individual primers, significant changes in shear bond strength before and after thermocycling occurred when using Alloy Primer (*p* = 0.019), the MKZ Primer (*p* < 0.001) and ArtPrime Plus Primer (*p* = 0.005) (Table 3, Figure 2).

### 3.2. Adhesive Bond on Zirconia Base

**Figure 3:**
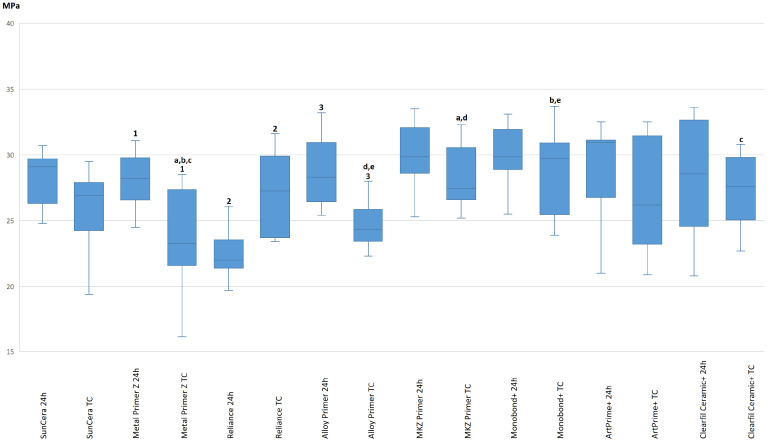
Comparison of the adhesive bond strength on zirconia surface (24 h versus thermocycling (TC)). Significant differences within a primer (24 h vs. TC) are indicated by Arabic numerals. Significant differences between different primers are indicated by lowercase letters. Equal numbers or lowercase letters indicate significant differences.

Table 4 summarizes the median and mean values determined before and after thermocycling on a zirconia base. After 24 h of water storage, bond strength values were in the range of 22.63 ± 2.28 MPa (Reliance Primer) to 29.96 ± 2.37 MPa (MKZ Primer). After thermocycling, the bond strength values ranged from 23.77 ± 3.86 MPa (Metal Primer Z) to 28.88 ± 3.09 MPa (Monobond Plus). Within the primers, the mean shear bond strength values after thermocycling changed significantly for Metal Primer Z (*p* = 0.011), Reliance Primer (*p* = 0.001) and Alloy Primer (*p* = 0.001) (Figure 3). While Metal Primer Z and Alloy Primer showed a reduction in bond strength after thermocycling, Reliance Primer was the only product that showed an increase in bond strength after thermocycling (Table 4).

Despite close bond strength ranges, statistically significant differences were seen between the different primers when used on a zirconia base (Kruskal–Wallis test *p* = 0.015). Significant differences were found between Metal Primer Z and MKZ Primer (*p* = 0.007), Monobond Plus (*p* = 0.002) and Clearfil Ceramic Primer Plus (*p* = 0.043) (Table 5, Figure 3). The aforementioned primers achieved significantly higher bond strengths on zirconia bases than Metal Primer Z. There was also a significant difference between Alloy Primer and MKZ Primer (*p* = 0.009) and Monobond Plus (*p* = 0.003), which also achieved significantly higher bond strengths than Alloy Primer (Table 5, Figure 3). All other comparisons between the primers were not statistically significant (Table 5).

### 3.3. Differences in Shear Bond Strength between BMA and Zirconia Base

**Figure 4:**
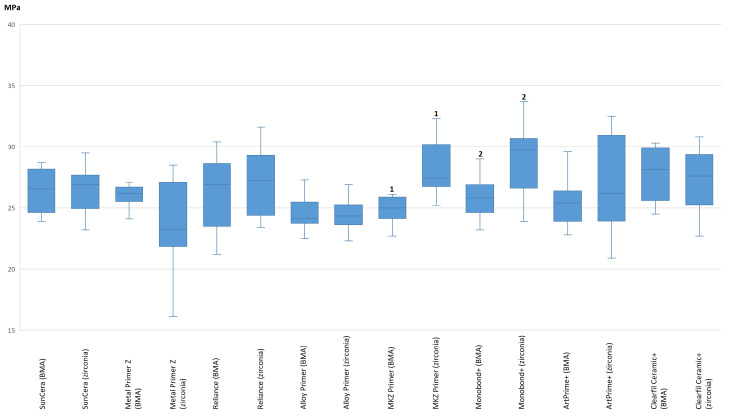
Comparison of the bond strength of the individual primers on a BMA vs. zirconia base. The boxplot graphs shown refer to the average bond strength after thermocycling. Significant differences between a primer on BMA and zirconia base are shown with Arabic numerals. Equal numbers indicate a significant difference.

Table 6 shows the mean differences between the BMA shear bond strengths and the zirconia shear bond strengths before and after artificial aging for each primer. After 24 h, with two exceptions, no statistically significant difference in the bond strength of the primers was observed for most of the primers when applied on either BMA or zirconia bases (maximum difference −1.59 MPa for Metal Primer Z). Significant differences after 24 h were found for Monobond Plus (−3.32 ± 2.20; *p* = 0.003) and Reliance Primer (+6.20 ± 4.91 MPa; *p* < 0.001). This did not change after thermocycling. In addition, the difference in the bond strength of the primers between BMA and zirconia was not significant, with two exceptions (maximum difference +2.35 MPa for Metal Primer Z). Significant differences in the application of the same primers on different base materials, however, were observed with Monobond Plus (−2.99 ± 3.83 MPa; *p* = 0.023) and with MKZ Primer (−3.08 ± 2.75 MPa; *p* = 0.001) (Figure 4).

### 3.4. Descriptive Analysis of Fracture Mode

**Figure 5:**
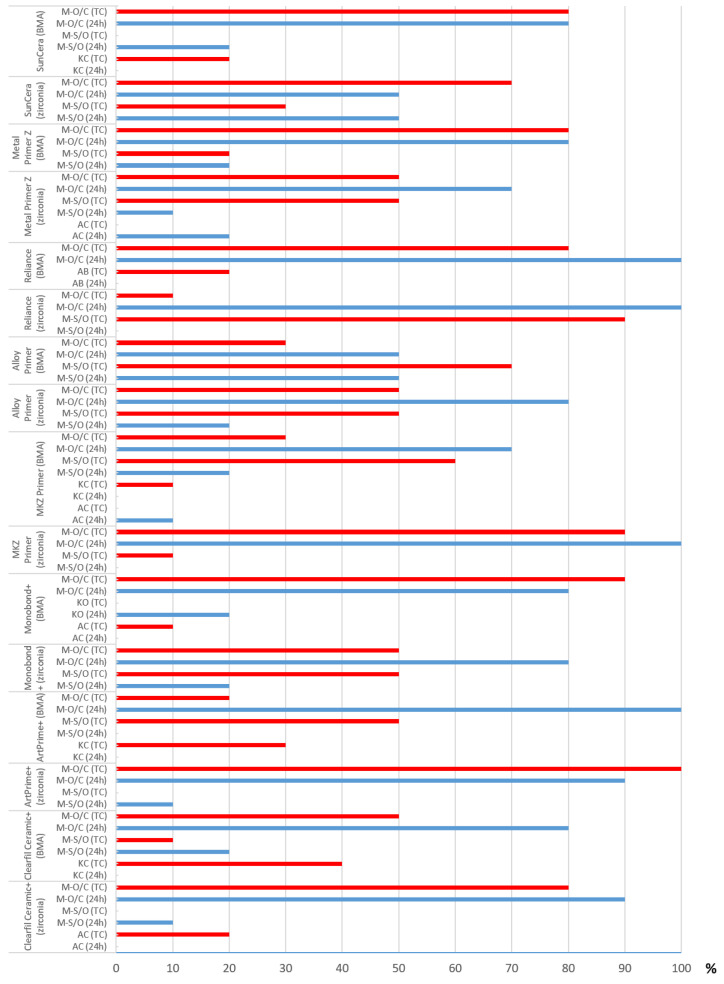
Evaluation of fracture mode after shear bond strength test. Red bar = proportion of corresponding fracture mode after thermocycling (TC), blue bar = proportion of corresponding fracture mode after 24 h water storage (24 h). AB = adhesive fracture on level of substrate base, AC = adhesive fracture on level of composite buildup, KO = cohesive fracture within the opaque, KC = cohesive fracture within the composite buildup, M-S/O = mixed fracture on level substrate/opaque, M-O/C = mixed fracture on level opaque/composite.

**Figure 6:**
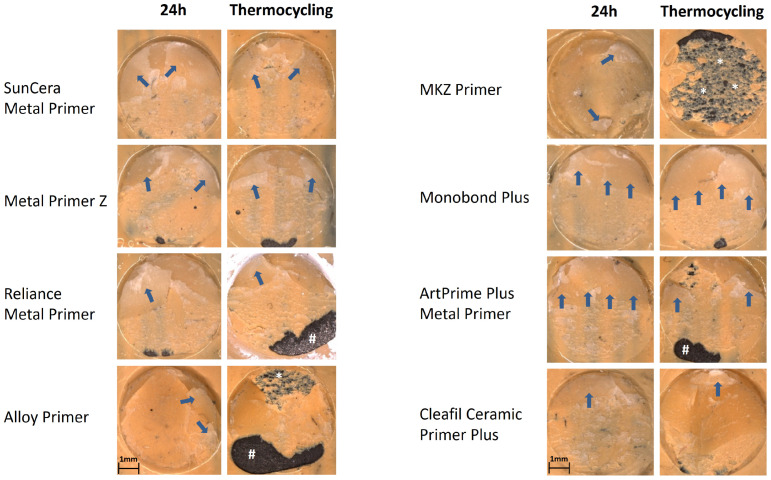
Representative stereomicroscopic images of the fracture surfaces on the BMA surfaces (Zeiss Axiotech microscope, Zeiss, Jena, Germany), image taken with Zeiss AxioCamMR5 (Zeiss, Jena, Germany) at 20× magnification). The blue arrows (**⇨**) mark remaining residues of the composite buildups. Areas marked with hashes (#) demonstrate areas with regional adhesive fractures on the BMA base. Areas marked with asterisks (*) show regions with mixed fracture on level substrate/opaque.

**Figure 7:**
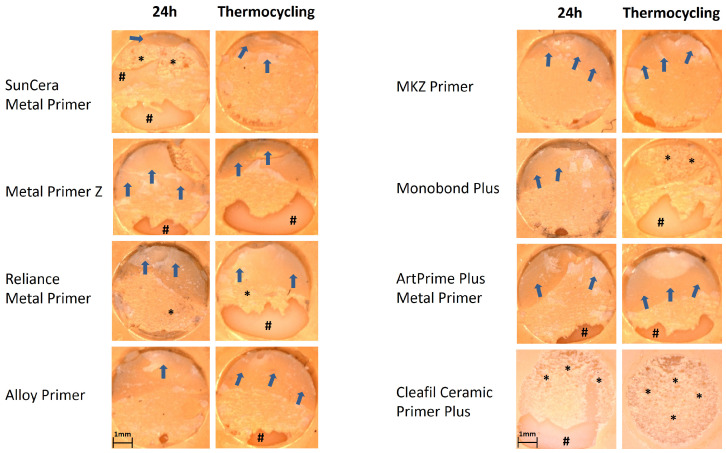
Representative stereomicroscopic images of the fracture surfaces on the zirconia surfaces (Zeiss Axiotech microscope, Zeiss, Jena, Germany), image taken with Zeiss AxioCamMR5 (Zeiss, Jena, Germany) at 20× magnification. The blue arrows (**⇨**) mark remaining residues of the composite buildups. Areas marked with hashes (#) demonstrate areas with regional adhesive fractures on the zirconia base. Areas marked with asterisks (*) show regions with mixed fracture on level substrate/opaque.

Microscopic examination of the fracture surfaces revealed mixed fractures in the area between the opaque and composite structure in most cases on both framework materials and storage conditions (Figure 5, Figure 6 and Figure 7). Mixed fractures occurred second most frequently between the material base and the opaque layer. Adhesive and cohesive fractures were very rare and only occurred sporadically within the series of measurements and were seen more frequently on BMA than on zirconia. On the BMA base, cohesive fracture within the composite buildup was the third most common fracture pattern after thermocycling. This occurred in 40% of the specimens when using the Clearfil Ceramic Primer, in 30% when using ArtPrime Plus and in 20% when using SunCera Primer, as well as in one case (10%) when using the MKZ Primer (Figure 5). On a zirconia base, only two cases (20%) of adhesive fractures were seen and were associated with Metal Primer Z (after 24 h) and Clearfil Ceramic primer (after thermocycling), all other specimens exhibiting mixed fractures.

## 4. Discussion

The aim of this study was to investigate whether modern one-bottle primers can reliably be applied to various framework materials. The study focused on the adhesive bond to BMA and zirconia, which are almost universally applicable and frequently used framework materials.

In general, the foundation of a secure adhesive bond between two materials depends on the good wettability of the substrate surface. The smaller the water contact angle, the better the surface can be completely wetted by the adhesive. A surface with as little water contact angle as possible is easier to wet with liquids [16,30]. Surface wetting can be improved by increasing the surface energy of the substrate base. This can be achieved on dental alloys, as well as on zirconia, by pretreatment using airborne-particle abrasion with aluminum oxide [31,32]. For zirconia, in particular, it has been well documented that surface airborne-particle abrasion with aluminum oxide (AL_2_O_3_) can increase the surface free energy and thus positively influence the adhesive bond [31,33,34].

The adhesive bond between the metal and composite is based on two synergy effects, including a mechanical and a chemical bonding component. Airborne-particle abrasion of the metal surface with AL_2_O_3_ has proven to be a very effective surface preparation technique [19]. On the one hand, the airborne-particle abrasion roughened surface improves micro retention, while the increased metal surface area offers more contact surface to the adhesive [9,19].

Primers containing acidic monomers with phosphoric or carboxylic groups have proven successful in achieving stable adhesive bonds between the metal base and the composite veneer [16,25]. The acidic groups ionize to form oxygen anions [20], interacting with the metal ions; thus, the acidic primers form a stable acid-base reaction [19]. It has been revealed by Suzuki et al. that 10-MDP, as one of the most relevant adhesion-promoting molecules in acidic primers, can be adsorbed by chromium. This explains the good adhesive properties of base metal alloys based on Co-Cr [35]. Ohno et al. investigated the adhesion mechanisms between functional monomers and Co-Cr alloy surfaces at a molecular level. Co-Cr alloys have a passive film on their surface, also known as hydrated chromium oxy-hydroxides, which has an amorphous layer thickness of 20–30 Å. The superficial areas of this film consist mainly of Co^2+^, while Cr^3+^ increases in the deeper layers. In the superficial layers, each Co and Cr molecule is surrounded by six OH and/or H_2_O groups. In the deeper layers, this ratio decreases, and the allocation is less than six. The authors concluded from their investigations of the adhesion of primers to Co-Cr alloys that three adhesion mechanisms between the functional primer monomers and the metal surface are most likely to occur: first, a primary atomic bond with the Co and Cr atoms; second, hydrogen bonding with the –OH groups of the passive film; and third, Van der Waals forces to hydrated molecules of the passive film [36].

Despite some similarities, the adhesion mechanisms based on zirconia differ from those based on BMA at the molecular level. For a sufficient bond between zirconia and resin composites, a reliable chemical bond has been proven to be achieved by conducting a combination of sandblasting of the zirconia surface and applying a phosphate-containing primer as well [31]. Primers containing the phosphoric acid monomer 10-MDP, in particular, have proven to be significantly advantageous for a reliable bond [4,17,31,37]. The bonding mechanism of 10-MDP on zirconia is based on ionic bonds, as well as on hydrogen bonds. During the chemical reaction of the 10-MDP with the zirconia surface, the phosphate-containing functional end of the 10-MDP dissociates. An ionic bond is formed between the negatively charged dissociated P-O^−^ group of the 10-MDP and the partially positively charged Zr^+^ ions of the zirconia surface. However, the main adhesive bond is formed by covalent hydrogen bonds between the phosphate oxygen groups (P=O) and the zirconium hydroxide group (Zr-OH) [33,38]. It is stated that a single 10-MDP monomer can bind both ionically and covalently to the zirconia surface via dissociation of the P-OH group and via the oxo group P=O [38]. If the concentration of 10-MDP is high enough, interactions between two adjacent 10-MDP monomers via the P=O of one monomer and the P=OH group of the adjacent monomer have also been described [38]. On the other hand, while a stable adhesive bond could also be generated on the BMA base with a silicatization and silanization layer, this kind of adhesive layer failed on the zirconia base [39].

In order to classify the adhesive bond values achieved in the present study, the German Institute for Standardization’s DIN EN ISO 10477 [26] was used as a benchmark. In accordance with the DIN standard, a bond strength of at least 5 MPa after the shear bond strength test must be archived. However, it remains critical to assess whether 5 MPa is a sufficient threshold for everyday clinical use. Behr and colleagues calculated a required bonding force of around 10 MPa for a dental restoration in the anterior region, taking into account the average masticatory force [40,41]. A review by Raszewski et al. even reports a target adhesive bond strength for secure adhesive cementation of fixed dental restorations in the range of 20 to 30 MPa [16]. The present study provides an indication that 10-MDP functions as an elementary bonding agent for a stable bond between BMA or zirconia and a veneering material. According to the manufacturer’s specifications or information in the literature where manufacturer details were missing, five out of the eight primers investigated in the present study contained 10-MDP. Nevertheless, in the present study, significant differences in the bond strengths among the 10-MDP-containing primers were seen. This could be explained by the different concentration of the 10-MDP monomers within the respective primers. It was described that the adhesive bond increases with increasing 10-MDP content of the priming agent [38,42]. A recent systematic review conducted by Ajay et al. [25], dealing with the bond strength of adhesive primers to metal alloys, confirms the need for such primers to significantly increase the bond strength. Among others, the authors included five studies in the systematic review, of which all investigated the bond strength of primers containing 10-MDP to Co-Cr alloys. Furthermore, all cited studies used thermocycling as an artificial aging method. The stated bond strength values after the shear bond strength test were 21.8 MPa (metal base with PMMA build-up; all specimens failed at the alloy/PMMA interface) [43], 28.6 MPa (metal base with PMMA build-up; all fractures were mixed fractures) [44], 30 MPa (metal base with composite build-up; all specimens failed at the alloy/opaque resin interface) [45], 41.1 MPa [46], and 43.4 MPa [47]. Yoshida et al. [46] and Matsumura et al. [47] bonded two Co-Cr alloy disks together for their adhesive bond tests and subjected them to shear bond strength tests. Matsumura et al. found cohesive fractures within the adhesive layer in seven out of eight cases, and Yoshida et al. [46] revealed in their study that all specimens failed cohesively within the adhesive layer. Fracture through the sheared buildup materials were not possible in both studies due to the selected Co-Cr structures, which explains the high adhesion values compared to the present study. At the same time, these studies demonstrate the high bonding potential of the 10-MDP-containing primers used.

Papadogiannis et al. [48] investigated the bond strength of universal adhesives on BMA and zirconia bases. The authors found that the universal adhesives examined on a Co-Cr surface outperformed the zirconia base. All universal adhesives used contained 10-MDP, and the authors attributed a decisive role to 10-MDP in the bonding process. However, no thermocycling was carried out in this study. Furthermore, the study cannot be directly compared with the present investigation, as it is unclear what influence the co-monomers of the universal adhesives have in comparison with the use of primers [48]. Sanohkan et al. used Alloy Primer and Monobond Plus, among others, for bond strength tests between zirconia and a composite resin buildup [18]. After 24 h storage in 100% humidity, they determined a shear bond strength of 16.8 MPa and 16.6 MPa, respectively, which was lower than the bond strength values determined in the present study for the same primers. Sanohkan et al. did not carry out any thermocycling either [18].

The fracture pattern analysis of the present study showed that most of the specimens exhibited a mixed fracture pattern with fracture progression on levels of opaque and composite. For both, BMA as well as zirconia bases, this type of fracture pattern occurred in around 70% of all shear bond strength tests carried out. The second most common fracture pattern was the mixed fracture at the level of the BMA or zirconia base and the opaque. Fractures at this level occurred in around a fifth of the BMA-based test specimens and a quarter of the zirconia-based test specimens. In around 10%, one of the other fracture forms occurred on the BMA base and in only 2.5% on the zirconia base. As can be seen in Figure 6 and Figure 7, mixed fractures frequently occurred, and only a small proportion of these penetrated through to the BMA or zirconia framework base. Therefore, the present study proves a secure bond at the level of the primer in the majority of cases. Based on this finding, it could be assumed that the primers used may have an even higher resistance to shear forces compared to composite resin. The fracture patterns show that a secure bond has formed between the butanediol-dimethacrylate-containing opaque and the urethane-dimethacrylate-based veneering composite. In addition to the methacrylate base, the opaque layer also contains light-blocking opaque fillers, which are intended to mask the metallic base and thus enable an esthetic tooth-colored buildup. The manufacturer of the opaque used in the present study recommends applying the opaque thinly with a brush and repeating the thin application after light curing until the desired coverage of the base has been achieved. Due to the strong light-blocking properties of the opaque, there is an increased risk that if the opaque layer is applied too thickly, the photosensitive polymerization initiators can no longer be sufficiently exposed, resulting in areas within the opaque layer that are insufficiently polymerized. The microscopic examination of the fracture surfaces in the present study indicated sufficient light polymerization. However, an opaque layer that is too thick before light curing must be considered critically in cases where a predominantly cohesive fracture in the opaque is observed, with simultaneously low adhesive bond strength. In the present study, all combinations of primers and material base were able to significantly exceed the bond strength threshold of 5 MPa, according to DIN EN ISO 10477 [26]. Even after stress tests using thermocycling, all combinations exceeded the required bond strength by a factor of five to six. In addition, all primers tested were within a low difference range. On the BMA base, the different primers did not differ significantly from each other after thermocycling and varied in a range of up to ±3 MPa. On the zirconia base, the difference range was slightly higher and significant, but within a manageable range of up to ±5 MPa.

The first null hypothesis, that there are no differences in the bond between the BMA base and the veneering composite when different common primers are used, can thus be accepted. The second null hypothesis, that there are no differences in the bond between the zirconia base and the veneering composite when different common primers are used, can only be partially accepted, since significant differences between the primers were found in individual cases. The comparison of the bond strength when using the primers on the two material bases showed a variation of up to ±3 MPa after thermocycling. Although the mean bond strength values were close to each other, two primer products differed significantly in the bond strength values when applied on BMA and zirconia base. The third hypothesis, that there are no differences in the determined bond between the BMA base and the zirconia base when different primers are used, can therefore only be partially accepted. As a clinical consequence, it can be concluded from the present study that all the one-bottle primers investigated were able to achieve a high degree of adhesive bond, both on the BMA and on the zirconia base. All the primers investigated thus meet the requirement for an efficient and universally applicable primer.

When assessing the results of this in vitro study, the strengths and limitations of the study should be identified and taken into account. One of the strengths of the present study is that it provides a broad overview of the performance of well-established and frequently used one-bottle primers. Another strength of the study that we emphasize is its study design, which corresponds exactly to the respective manufacturer’s specifications and can thus be applied with a high degree of validity to the application in a dental laboratory. It can be assumed that, despite the in vitro study set-up, the bond strength values determined are reproducible when used in a dental laboratory. In addition, the data generated in the present study can be used as a database and reference for the investigation and classification of newly developed primers. Besides the strengths, however, the present study also has some limitations. While the data obtained can be transferred very well to the situation in a dental laboratory, these laboratory conditions are not necessarily comparable with the situation in the oral cavity, since the conditions in vivo, i.e., within the oral cavity, may be completely different. This becomes relevant to the point where the presented procedure is to be applied, in the case of an intraoral ceramic repair, on a fixed dental restoration such as a crown or bridge suffering from a ceramic chipping. The moist environment of the oral cavity can lead to significantly lower bond strength values if the area to be repaired is not accurately drained [16]. This must be considered when interpreting and classifying the bond strength values. Ultimately, a fundamental limitation of exploratory studies is that they can only provide an overview of a specific field and that it is never possible to represent all possible combinations in a single study. Nevertheless, studies like the present one can provide certainty if the functional application of the primers used in everyday life is confirmed.

## 5. Conclusions

The present exploratory in vitro study was able to show that modern one-bottle primers can generate a reliable bond between a BMA base and a composite resin buildup, as well as between a zirconia base and a composite resin buildup. Even if the adhesive bond appears to be more consistent with the BMA base, the primers examined also achieved solid adhesive bond strength values with the zirconia base, despite some statistical differences. The phosphoric acid bifunctional monomer 10-MDP appears to have a decisive influence on a sufficient adhesive bond. While the results can be applied almost without restriction to processing in a dental laboratory due to the selected study design, the changed environmental conditions in the moist oral cavity must be considered when applying the primers.

## Figures and Tables

**Table 1:** Technical data of opaque and composite used for specimen preparation.

Primer	Functional Components	Application/Polymerization	Batch No.
ArtPreOpaque Plus (Merz Dental, Luetjenburg, Germany)	Methacrylic-ester	Use after application of a metal primer. Apply thinly using a brush with short, stiff bristles, light curing time depending on the polymerization unit used: 60–300 s	2019006411
ArtOpaque Plus O1 (Merz Dental, Luetjenburg, Germany)	BDDMA, TPO	Apply twice thinly using a brush with short, stiff bristles until the framework is completely covered with color, light curing time depending on the polymerization unit used: 60–300 s	2019009066
Ceramage Body light curing crown and bridge composite (A2B) (Shofu, Kyoto, Japan)	UDMA, UDA, zirconium silicate, pigments, others	Final light curing 180 s	112045

BDDMA = Butandiol-dimethacrylate; TPO = diphenyl(2.4.6-trimethylbenzoly)phosphin-oxide; UDMA = Urethane dimethacrylate; UDA = 2-Uretdione diamide.

**Table 3:** Shear bond strength values (MPa) between the BMA base and the veneering composite when using different primers.

		Median	IQR	Mean (SD)	95%CI	*p*-Value
SunCera Metal primer	24 h	28.75	5.3	29.12 (3.91)	26.32–31.92	0.143
	TC	26.55	4.0	26.89 (3.10)	24.67–29.11	
Metal Primer Z	24 h	25.45	5.6	26.53 (3.28)	24.18–28.88	0.971
	TC	26.20	1.6	26.12 (1.30)	25.19–27.05	
Reliance	24 h	28.60	5.7	28.83 (3.22)	26.53–31.13	0.165
	TC	26.90	6.2	26.16 (3.22)	23.85–28.47	
Alloy Primer	24 h	27.80	4.9	27.87 (3.37)	25.46–30.28	0.019
	TC	24.15	2.7	24.72 (1.83)	23.41–26.03	
MKZ Primer	24 h	30.10	2.9	29.72 (2.00)	28.29–31.15	<0.001
	TC	25.00	1.9	25.19 (1.73)	23.95–26.43	
Monobond Plus	24 h	26.75	4.4	26.58 (2.52)	24.78–28.39	0.436
	TC	25.80	3.1	25.89 (2.00)	24.46–27.32	
Art Prime Plus	24 h	29.05	2.5	28.78 (1.82)	27.48–30.08	0.005
	TC	25.40	3.3	25.62 (2.31)	23.97–27.27	
Clearfil Ceramic Plus	24 h	29.65	7.2	29.33 (4.03)	26.45–32.21	0.315
	TC	28.15	4.8	27.69 (2.37)	26.00–29.38	

Comparison of shear bond strength after 24 h of water storage and after 25,000 thermocycles (TC) using the Mann–Whitney U test.

**Table 4:** Shear bond strength values (MPa) between the zircon base and veneering composite when using different primers.

		Median	IQR	Mean (SD)	95%CI	*p*-Value
SunCera Primer	24 h	29.10	3.4	28.21 (2.00)	26.78–29.64	0.075
	TC	26.90	3.6	26.03 (2.96)	23.91–28.15	
Metal Primer Z	24 h	28.20	3.2	28.12 (2.03)	26.67–29.57	0.011
	TC	23.25	5.8	23.77 (3.86)	21.01–26.53	
Reliance Primer	24 h	22.00	2.2	22.63 (2.28)	21.00–24.26	0.001
	TC	27.25	6.2	27.22 (3.06)	25.03–29.41	
Alloy Primer	24 h	28.30	4.5	28.80 (2.66)	26.90–30.70	0.001
	TC	24.35	2.4	24.66 (1.72)	23.43–25.90	
MKZ Primer	24 h	29.85	3.5	29.96 (2.37)	28.27–31.66	0.123
	TC	27.45	3.9	28.27 (2.29)	26.63–29.91	
Monobond Plus	24 h	29.85	3.0	29.90 (2.18)	28.34–31.46	0.684
	TC	29.75	5.4	28.88 (3.09)	26.67–31.09	
ArtPrime Plus	24 h	30.95	4.4	28.52 (4.78)	25.10–31.94	0.579
	TC	26.20	8.2	27.00 (4.09)	24.08–29.92	
Clearfil Ceramic Plus	24 h	28.55	8.1	28.39 (4.55)	25.14–31.64	0.481
	TC	27.60	4.8	27.26 (2.64)	25.37–29.15	

Comparison of shear bond strength after 24 h of water storage and after 25,000 thermocycles (TC) using the Mann–Whitney U test.

**Table 5:** Comparison of differences in shear bond strength on zirconia base when using different primers.

	SunCera Primer	Metal Primer Z	Reliance Primer	Alloy Primer	MKZ Primer	Mono-Bond Plus	ArtPrime Plus	Clearfil Ceramic Plus
SunCera Primer	x	0.201	0.510	0.231	0.156	0.071	0.637	0.459
Metal Primer Z	0.201	x	0.052	0.935	0.007	0.002	0.080	0.043
Reliance Primer	0.510	0.052	x	0.063	0.447	0.252	0.851	0.935
Alloy Primer	0.231	0.935	0.063	x	0.009	0.003	0.095	0.052
MKZ Primer	0.156	0.007	0.447	0.009	x	0.700	0.343	0.497
Monobond Plus	0.071	0.002	0.252	0.003	0.700	x	0.183	0.288
ArtPrime Plus	0.637	0.080	0.851	0.095	0.343	0.183	x	0.788
Clearfil Ceramic Primer Plus	0.459	0.043	0.935	0.052	0.497	0.288	0.788	x

Shown are *p*-values of the differences between the various primers on zirconia base. Underlying data are bond strength values after thermocycling from Table 4. *p*-values are determined by using the Kruskal–Wallis test.

**Table 6:** Difference of shear bond strength values between BMA and zirconia.

Primer	BMA vs. Zirconia			
	24 h		Thermocycling	
	Mean Diff (SD) [MPa]	*p*-Value	Mean Diff (SD) [MPa]	*p*-Value
SunCera Primer	0.91 (3.83)	0.971	0.86 (4.11)	0.912
Metal Primer Z	−1.59 (1.84)	0.218	2.35 (4.03)	0.280
Reliance Primer	6.20 (4.91)	<0.001	−1.06 (4.59)	0.436
Alloy Primer	−0.93 (3.54)	0.684	0.06 (0.99)	0.970
MKZ Primer	−0.24 (3.80)	0.853	−3.08 (2.75)	0.001
Monobond Plus	−3.32 (2.20)	0.003	−2.99 (3.83)	0.023
ArtPrime Plus	0.26 (4.91)	0.315	−1.38 (5.26)	0.529
Clearfil Ceramic Primer Plus	0.94 (4.45)	0.796	0.43 (2.44)	0.853

The mean average of the difference between MPa_(zirconia)_–MPa_(BMA)_ is shown. A Mann–Whitney U test was used to determine the statistical significance in the mean difference.

## Data Availability

The data are not publicly available as parts of the data are part of an ongoing doctorial thesis. Requests to access the datasets could be directed to the corresponding author.
